# P360: Materiovigilance and improvement of the maintenance of the biomedical equipment by the implementation of strategies for the use of equipment: case study of the hospital Gabriel Touré of Mali

**DOI:** 10.1186/2047-2994-2-S1-P360

**Published:** 2013-06-20

**Authors:** T Dieffaga, M Sanogo, S Maiga

**Affiliations:** 1 Hospital Gabriel Touré, Bamako, Mali; 2 Medecine Faculty, School of Public Health, University of Montreal , Montreal, Canada; 3 Faculty of Medicine, Pharmacy and Dentistry, Bamako, Mali

## Objectives

To make the inventory of current practices for the use of the large equipment at the hospital Gabriel Touré and to bring solutions for an improvement of the use and productivity of the equipment.

## Methods

This was a cross-sectional study using the method of problem resolution by quality improvement. It was conducted in 2004in two engineering departments of the hospital Gabriel Touré. 38 users of the equipment were concerned with the investigations.

## Results

Of 38 surveyed persons only two doctors could operate the echograph. 3 persons of 38 (7.89%) were abele to select the right parameters for the use of the equipment. Test of use is not carried out by 20 agents (52.63%), including 11 persons that manipulate equipment of sterilization and medical imagery. 14 agents carry out the act badly including 5 technicians’ that manipulate radiograpies what explains the important loss of simages. Only 8 agents (21.05%) turn the equipment off after work. 18 agents do not carry out maintenance of the material after use including 9 users of radiography and echography.

## Conclusion

The problem of maintenance at the hospital Gabriel Touré is a crucial question. The constraints related to the management of the large equipment at the hospital Gabriel Touré relate to several spects: the deficit of information, the absence of supervision and framing, the insufficiency of sensibilzation, the deficit of the actors implied in information and adapted know-how, the weakness of financial means and material allocated, the insufficiency of clear and codified directives fixing the conditions of use of the equipment. We recommend an internal reorganization of the service of maintenance, the programming of regular supervision, the development and the implementation of the management tools of maintenance.

## Disclosure of interest

None declared

